# Lymphoepithelial cyst mimicking malignant pancreatic signs: a case report

**DOI:** 10.1186/s13256-023-04087-6

**Published:** 2023-08-21

**Authors:** Christian Teske, Jürgen Weitz, Frieder Meier, Jens-Peter Kühn, Carina Riediger

**Affiliations:** 1 Department of Visceral, Thoracic and Vascular Surgery, University Hospital Carl Gustav Carus Dresden, Fetscherstrasse 74, 01307 Dresden, Germany; 2 National Center for Tumor Diseases (NCT/UCC), Dresden, Germany; 3 Department of Pathology, University Hospital Carl Gustav Carus, Dresden, Germany; 4 Department of Radiology, Institute of Diagnostic and Interventional Radiology, University Hospital Carl Gustav Carus, TU, Dresden, Germany; 5 German Cancer Research Center (DKFZ), Heidelberg, Germany; 6 Faculty of Medicine, University Hospital Carl Gustav Carus, Technische Universität Dresden, Dresden, Germany; 7 Helmholtz-Zentrum Dresden-Rossendorf (HZDR), Dresden, Germany

**Keywords:** Lymphoepithelial cyst, Pancreas, Cystic lesion, Case report

## Abstract

**Background:**

A lymphoepithelial cyst of the pancreas is a rare benign lesion that is difficult to diagnose preoperatively and challenging in distinguishing from potentially malignant cystic pancreatic neoplasms. A diagnostic step-up approach is recommended to clarify the lesion’s dignity and specify a treatment plan.

**Case presentation:**

Here, we describe a case of a 51-year-old male European with a lymphoepithelial cyst of the pancreas mimicking malignant features in a mid-age male patient with abdominal pain and unintended weight loss.

**Conclusion:**

Patients with indeterminate cystic pancreatic lesions should be examined by a multidisciplinary diagnostic team in a step-up approach to clarify the lesion’s entity. In the case of incidentally found lymphoepithelial cysts of the pancreas, a watchful waiting strategy might be clinically reasonable if the diagnosis is proven.

## Introduction

Pancreatic cystic lesions are a heterogenous group of pancreas-related entities with a rising detection rate due to recent imaging improvements [1, 2]. The differentiation between neoplastic lesions with a risk of malignant transformation and non-neoplastic lesions is crucial for clinical management, but often challenging concerning various classification systems. Neoplastic lesions include intraductal papillary mucinous neoplasms (IPMN), mucinous and serous cystic neoplasms and solid pseudopapillary neoplasm among others [3]. Whereas non-neoplastic cystic lesions without the risk of cancer development consist mainly of inflammatory cystic lesions such as pseudocysts but also cystic lymphangioma, retention cysts and lymphoepithelial cysts.

Preoperative workflow including several imaging modalities and fine needle aspiration (FNA) focuses on the specific diagnosis with subsequent treatment options. Some cases remain unclear with inconsistent results in histology, imaging and clinical presentation. In these patients, surgical resection of the lesion remains state of the art. In the following case, we report of an extremely rare entity of a lymphoepithelial cyst of the pancreas mimicking malignant preoperative features.

## Case history

A 51-year-old male European patient presented with undetermined epigastric pain and recurrent postprandial nausea. A history of recurrent mild episodes of pancreatitis treated by the general practitioner without the need of hospital admissions was notified during the last 4 years. The patient reported of unintended weight loss of 7 kg within 3 months. Proton-pump inhibitors were taken in double standard dosage without changing symptoms. A diabetes mellitus type II was first diagnosed 10 years ago with a need of insulin therapy for the last 3 years. Arterial hypertension, hypothyroidism and hyperlipidemia were sufficiently treated. The patient was smoker with a cumulative value of 30 pack years. Alcohol consumption was negated and exocrine pancreas function was not impaired. Family history of malignant neoplasm was unremarkable.

## Investigation and treatment

**Fig. 1:**
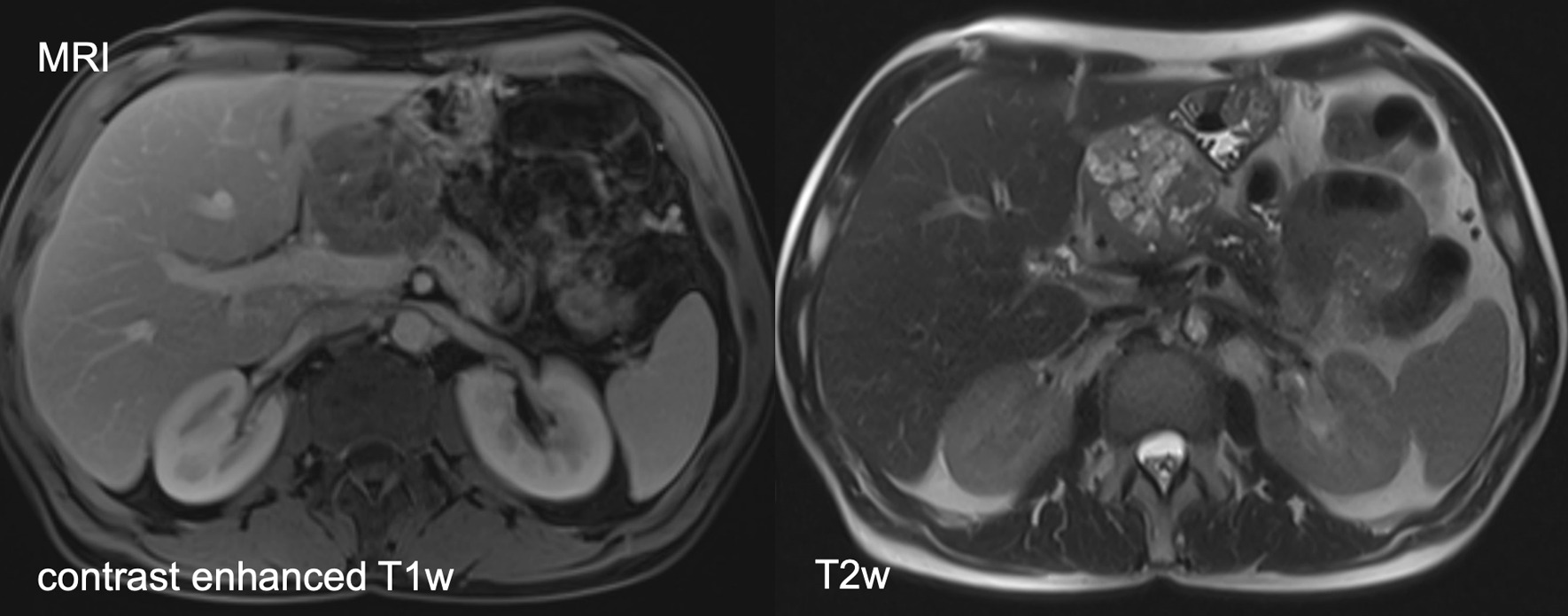
MRI revealing a 5 × 6 cm inhomogeneous polycystic mass with suspected solid components in the pancreatic head with a partly compression of the duodenum

**Fig. 2:**
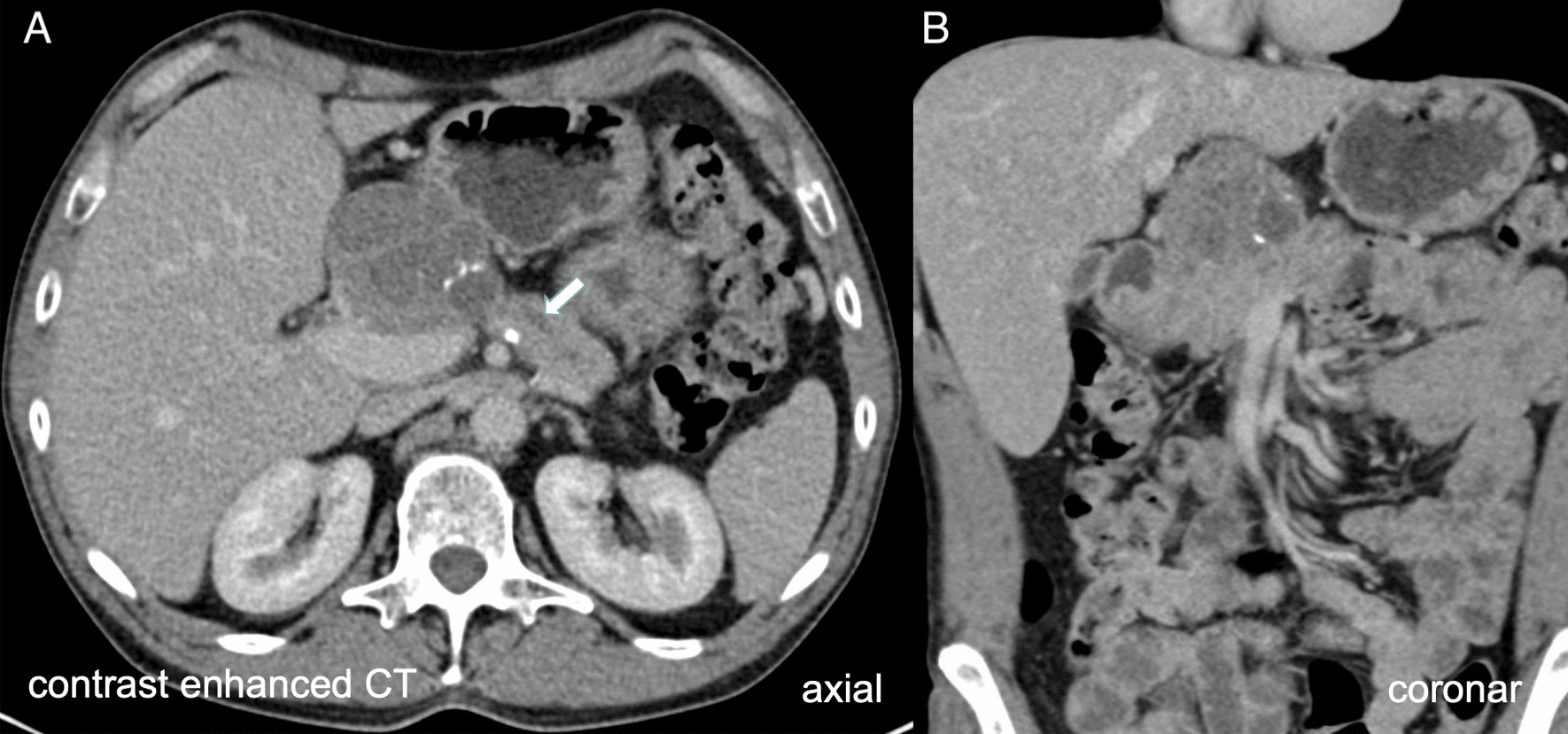
Contrast enhanced computed tomography (CT) scan revealed a septated cystic lesion with solitary calcification in axial (**A**) and coronar (**B**) view with potential infiltration of the duodenum. The pancreatic duct was shown dilated up to 6mm (arrow). The dignity of the lesion cannot be conclusively clarified

Due to the unintended weight loss an esophago-gastro-duodenoscopy and colonoscopy were performed displaying mild esophagitis I° and Helicobacter pylori-negative gastritis as well as sigma diverticulosis and an isolated colon polyp without any pathologic sign. Alterations of the duodenum were not recorded. As a step-up approach, the patient underwent an epigastric magnetic resonance imaging (MRI) revealing a 5 × 6 cm inhomogeneous, polycystic mass in the pancreatic head with a dilated pancreatic duct and a partly compression of the duodenum (Fig. 1). A further contrast enhanced computed tomography (CT) scan was initiated showing a potential infiltration of the duodenum and the left gastric artery, respectively (Fig. 2). The superior mesenteric vein and artery were regularly contrasted with no signs of infiltration. Isolated enlarged loco-regional lymph nodes were seen without any other distant spread.

Analysis of the blood displayed no pathologic parameter except for a minimally elevated CA 19–9 of 93.3 U/ml (cut off: < 34.0 U/ml), CEA was in normal range, pancreatic enzymes were not deflected.

Due to the young age and the minimal elevated tumor markers in the serum, an endoscopic ultrasound with fine needle aspiration (FNA) biopsy of the mass was performed. Ultrasound analysis supported the suspected malignant entity. Histology results showed necrotic tissue and normal small intestine mucosa without atypical cells.

Although FNA revealed no malignant cells, considering the infiltrative growth of the mass according to CT scan, a surgical resection (Whipple’s procedure) was performed. Intraoperatively, the peripancreatic region showed a massive inflammatory process and surgical dissection was highly challenging. The cystic mass was removed completely and fresh frozen section analysis revealed no tumor infiltration into the margin regions.

**Fig. 3:**
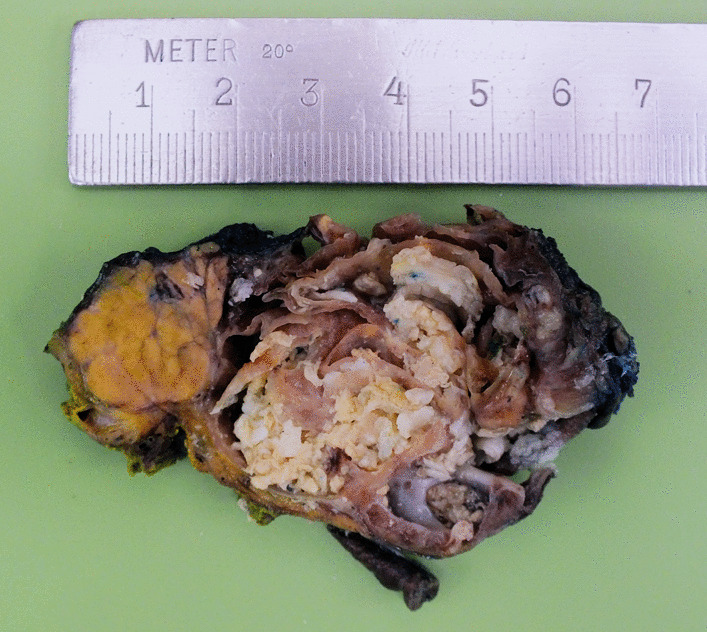
Macroscopic analysis of the resection specimen displays a cystic lesion with septation and crumbly content adjacent to the pancreas

**Fig. 4:**
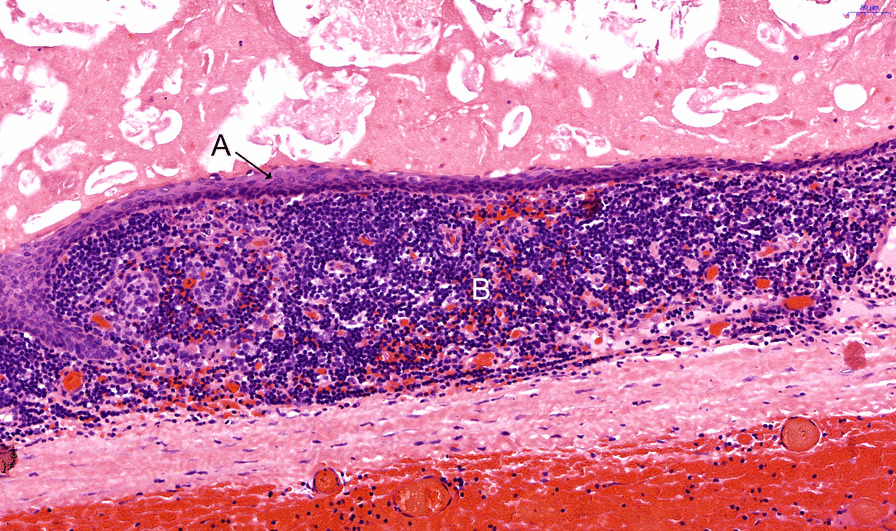
Hematoxylin and eosin-stained microscopic sample displays squamous epithelial cells (**A**) without atypical signs surrounded by lymphatic tissue (**B**)

**Fig. 5:**
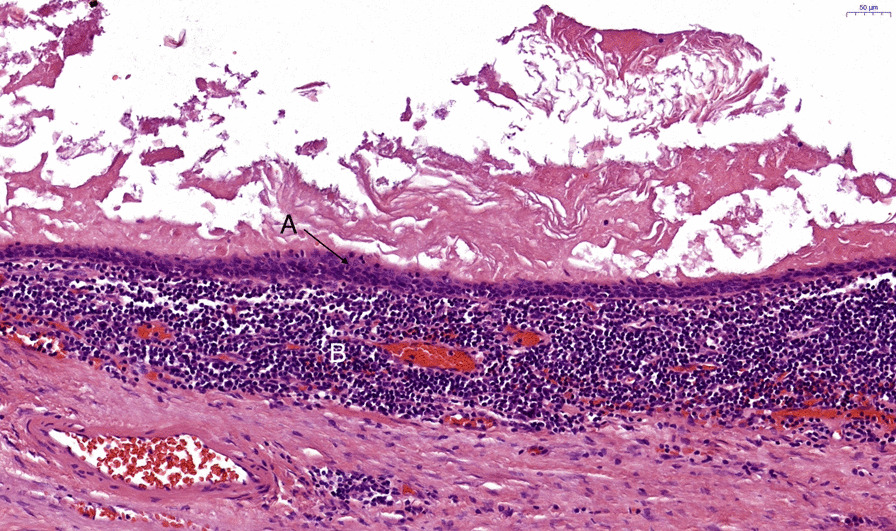
Hematoxylin and eosin-stained microscopic sample displays squamous epithelial cells (**A**) without atypical signs surrounded by lymphatic tissue (**B**)

The final histological analysis reported a lymphoepithelial cyst of the pancreas with exophytic growth regions. Macroscopically, a 6.5 × 5 × 3.5 cm cystic mass with white, crumbly, sebum-like content associated with several minor similar, multi-cystic lesions with the same content adjacent to the pancreas was seen, completely surrounded by a capsule-like border (Fig. 3). Microscopic analysis revealed multiple cystic lesions with squamous epithelium lacking atypical cells. Additionally, a few respiratory epithelial cells were detected surrounded by lymphatic tissue (Figs. 4 and 5). Low grade intraepithelial neoplastic regions of the pancreatic duct and highly active, partly purulent inflammatory processes were found. All resected lymph nodes revealed no malignant cells.

## Outcome

The postoperative course of the patient was characterized by a lymphatic fistula sufficiently managed by prolonged intravenous nutrition in combination with subcutaneous sandostatin therapy (ocreotide, 3 × 100 µg per day). Pain management was performed according to standard protocol without any excessive pain episodes. On postoperative day 16, the patient was discharged well-conditioned, pain-free with normal oral nutrition on pancreatic enzyme substitution. The foreknown insulin-dependent diabetes mellitus was managed equally compared to preoperative conditions. The one-year postoperative follow-up revealed no changes in the patient’s health condition.

## Discussion

Lymphoepithelial cyst of the pancreas is a rare, non-malignant diagnosis first described in 1985 by Lüchtrath and Schriefers [4]. The detailed pathogenesis of the lesion is, by date, not fully understood. Theories include remnants of epithelial cells within lymph nodes, metaplasia of pancreatic ductal cells and derivation from teratomas [5]. Although the microscopic criteria for a metastasis from a distant mature teratoma were not given in the present case, it has to be considered as differential diagnosis. Main clinical characteristics of lymphoepithelial cysts are a predominant occurrence in male, mid-age patients with unspecific abdominal and back pain as well as nausea. However, most of the cases are found incidentally by ultrasound or CT scans [5, 6]. According to the literature, there is no recurrence or malignant progression documented [6].

As in the present case, lymphoepithelial cysts of the pancreas are often mimicking criteria of malignancy in imaging such as dilatation and/or discontinuation of the pancreatic duct, infiltration of surrounding structures or vessels. Thus, potentially malignant cystic lesions of the pancreas, such as intraductal papillary mucinous neoplasms or mucinous cystic neoplasms, may be misdiagnosed. Due to the difficulties in reaching an accurate diagnosis preoperatively, most cases are surgically resected.

Management of cystic pancreatic lesions requires a patient-oriented selective approach to fulfill the diagnostic and therapeutic criteria [7]. Recent studies concluded that endoscopic ultrasound guided evaluation of a cystic pancreatic lesion was not sufficient in clarification the malignant potential if prior imaging results were inconsistent [8, 9]. Furthermore, a combination with FNA and a molecular analysis of the cystic fluid helps rising the diagnostic yield [10–12]. In a recent study, an additional KRAS mutational analysis of the FNA tissue sample increased the method’s sensitivity for the diagnosis of IPMN up to 92% [13]. Okasha and colleagues demonstrated a clear and practical overview about the diagnostic step-up approach with treatment indications in patients with pancreatic cysts [14]. In a first step, CT or MRI in combination with a MRCP is indicated in undetermined cystic lesions. Due to the rare appearance of lymphoepithelial cysts especially in the pancreas, there are no distinctive imaging features being specific for these entities.

If high risk stigmata are present (bile duct dilation, pancreatic duct ≥ 10 mm, enhancing mural nodule) and the patient is in good clinical shape, an upfront surgery is recommended. In surgically unfit patients, ablation methods should be considered in these cases. Apart from high-risk stigmata, there are worrisome features requiring further evaluation by endosonographic ultrasound and FNA. These features consist of a lesion diameter of more than 3 cm, pancreatic duct dilation of 5–9 mm, enhancing cystic wall, non-enhancing mural nodule and a suspicious lymphadenopathy. If neither high-risk stigmata nor worrisome features are present on imaging, a conventional follow-up is recommended after 6 months to 2 years depending on the current lesion size. The authors favor an initial re-imaging (CT) after 6 months to exclude misdiagnosed malignant lesions. Hereafter, an expansion of imaging surveillance may be reasonable. Unfortunately, there are no liquid biopsy markers for a better monitoring yet.

However, the present case shows the remaining striking difficulty in finding a sufficient preoperative diagnosis of cystic pancreatic lesions.

## Conclusion

In conclusion, patients with indeterminate cystic pancreatic lesions should be examined by a multidisciplinary diagnostic team. If the results of the diagnostic step-up approach remain unclear and the entity cannot be clarified, surgery should be favored. In the case of incidentally found small lymphoepithelial cysts of the pancreas in asymptomatic patients, a watchful waiting strategy might be clinically reasonable if the diagnosis is proven.

## Data Availability

Not applicable.
